# Supplementary material for: Small molecules as potent biphasic modulators of protein liquid-liquid phase separation
Source: Nat Commun. 2020 Nov 4;11:5574. doi: 10.1038/s41467-020-19211-z (PMC7643064; doi:10.1038/s41467-020-19211-z)
Supplement: Supplementary file 1 — Supplementary Information [file 41467_2020_19211_MOESM1_ESM.pdf]

SUPPLEMENTARY INFORMATION

Small molecules as potent biphasic modulators of protein liquid-liquid phase separation

W. Michael Babinchak[1], Benjamin K. Dumm[1], Sarah Venus[2], Solomiia Boyko[1], Andrea A. Putnam[2,3], Eckhard Jankowsky[2], and Witold K. Surewicz[1,*]

[1]Department of Physiology and Biophysics and [2]Center for RNA Science & Therapeutics, Case Western Reserve University, Cleveland, OH USA

[3]Present address: Department of Molecular Biology and Genetics, Johns Hopkins School of Medicine, Baltimore, MD USA

[*]Corresponding author. Email: wks3@case.edu

Table of Contents

1. Supplementary Table
2. Supplementary Figures

Supplementary Table 1. **Partition coefficients (logP) between polar and non-polar solvent attained from PubChem and ChemSpider.**

Compound	Experimentally-determined logP	Computed XlogP3 or XlogP3-AA	ChemSpider ID	PubChem CID
Adenosine triphosphate (ATP)	--	-5.7	5742	5957 (XLogP3)
Cyclic di-AMP	--	-5.3	9333199	11158091 (XLogP3-AA)
Adenosine cyclic 3',5'-monophosphate (cAMP)	--	-2.6	5851	6076 (XLogP3-AA)
Adenosine	-2.367*	-1.1	54923 (LN00194348)	60961 (XLogP3)
Adenine	-1.287*; -0.026#	-0.1	185 (LN00008093; STK387542)	190 (XLogP3)
Benzene sulfonate	--	-0.1	82647	91526 (XLogP3)
2-Naphthalene sulfonic acid	0.718*	0.6	8113 (LN00195536)	8420 (XLogP3)
8-anilino-1-naphthalene sulfonic acid (ANS)	0.952*	3.5	1328 (LN00194340)	1369 (XLogP3-AA)
1,1'-binaphthyl	6.202*; 5.1652&	6	11296 (LN00188221; SL063893)	11789 (XLogP3-AA)

Only compounds with logP values accessible via PubChem or ChemSpider are shown. Experimentally-determined logP values that were attained via ChemSpider come from the following sources: *LabNetwork (LN); #Vitas-M (STK); &Synthon-Lab (SL). Computed XlogP3 and XlogP3-AA (atom additive model) were attained via PubChem.

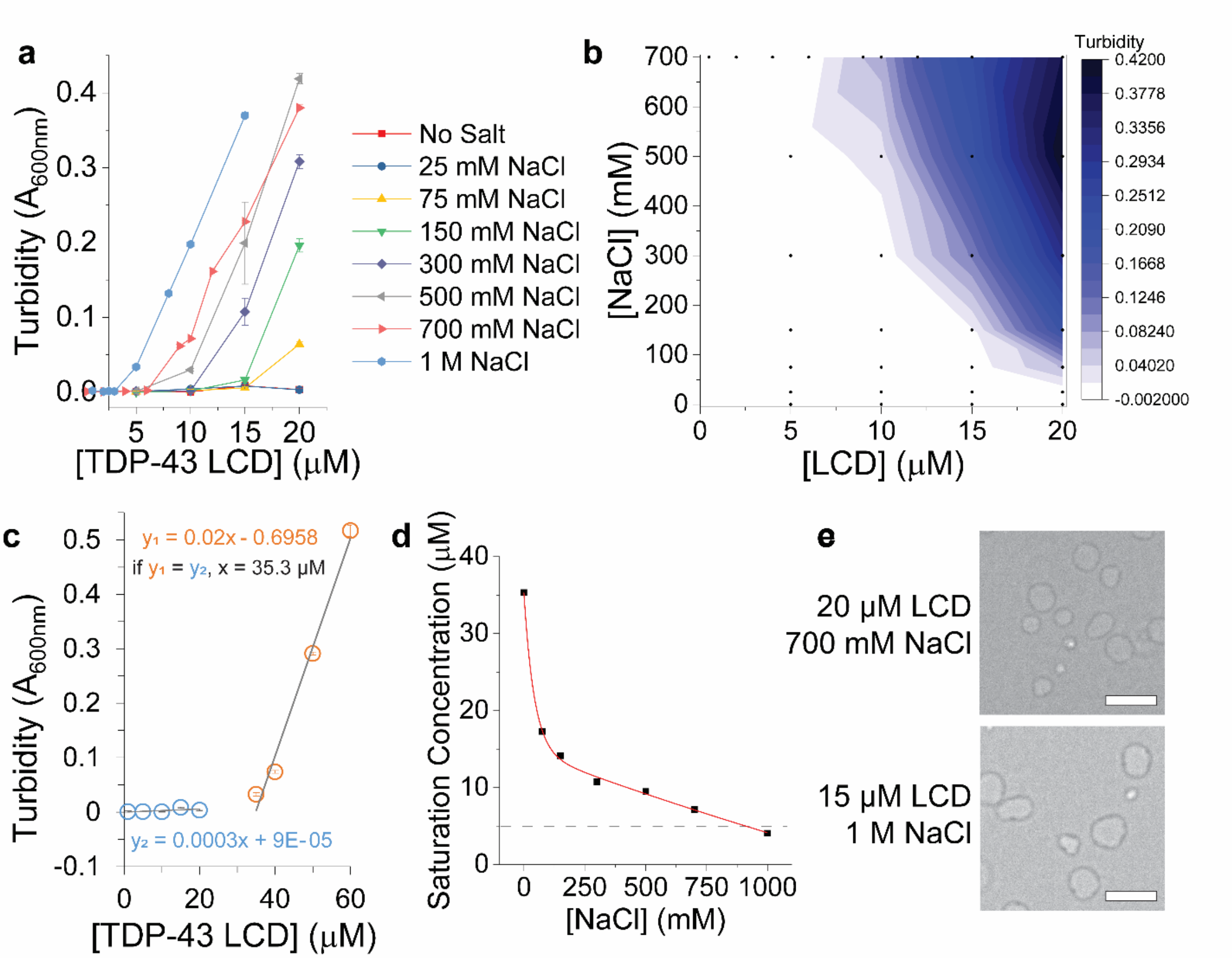

Supplementary Figure 1. **Characteristics of TDP-43 LCD homotypic LLPS in the presence of NaCl at pH 6. a,** Turbidity traces as a function of TDP-43 LCD concentration in the presence of varying concentrations of NaCl. **b,** Turbidity contour plot (phase diagram) depicting the relationship between NaCl and TDP-43 LCD concentrations. Data points (from panel **a**) used for contouring are shown in black. **c,** Representative calculation based on turbidity measurements to determine saturation concentration in the absence of NaCl. The blue line is drawn through data points indicating the absence of LLPS, while the orange line is drawn through data points in which robust LLPS occurs. The concentration of protein at which these two lines intersect is an estimation of the c_{sat}. **d,** Saturation concentrations as a function of NaCl concentration (determined as described in **c**). A horizontal dashed line is drawn to demonstrate that a c_{sat} for homotypic LLPS only reaches below 5 μM protein in the presence of extremely high concentrations of NaCl (1 M). **e**, Representative bright field microscopy images demonstrating the presence of droplets at high concentrations of NaCl. Experiments were performed in a 20 mM potassium phosphate buffer, pH 6.0. Scale bars: 10 μm. (*n* = 3 technical replicates). All data are presented as mean values +/- standard deviation.

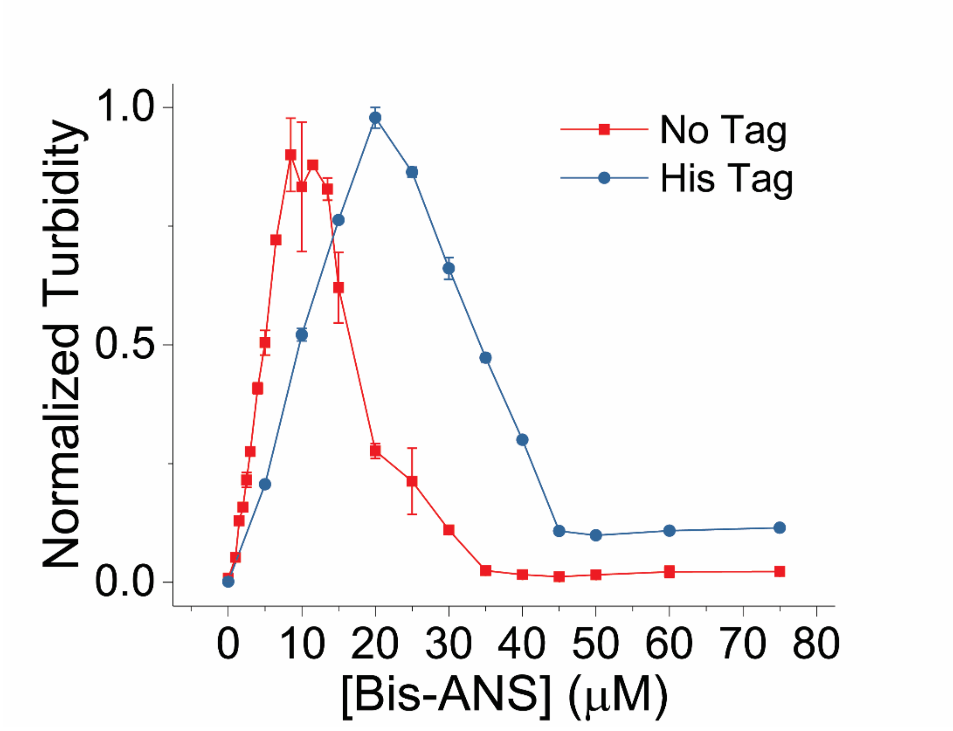

Supplementary Figure 2. **Effect of bis-ANS on LLPS of His-tag and untagged TDP-43 LCD.** Normalized turbidity as a function of bis-ANS concentration for TDP-43 LCD with and without His-tag (5 μM each). All experiments were performed in a 20 mM potassium phosphate buffer, pH 6.0. ($n \geq 3$ technical replicates across 3 separate preparations for untagged protein (this is normalized data from Fig. 1c), $n = 3$ technical replicates for tagged protein). All data are presented as mean values +/- standard deviation.

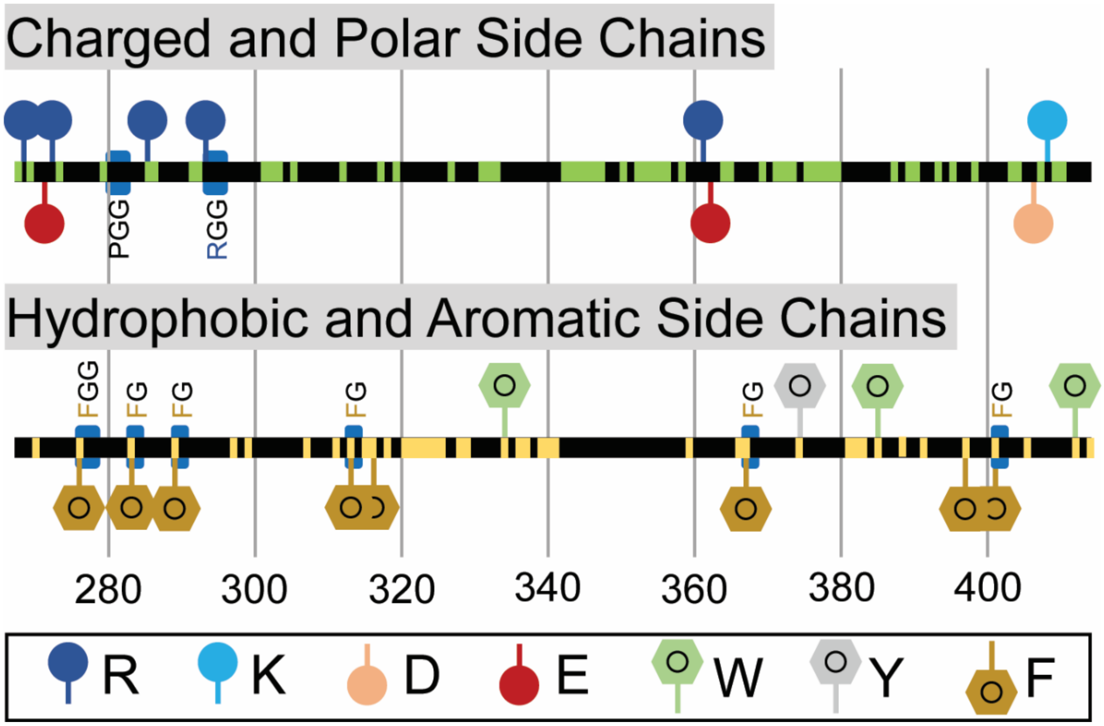

Supplementary Figure 3. **Schematic of TDP-43 LCD sequence**. Polar and hydrophobic amino acids are indicated by green and yellow bars, respectively. Charged and aromatic side chains are also depicted in the legend.

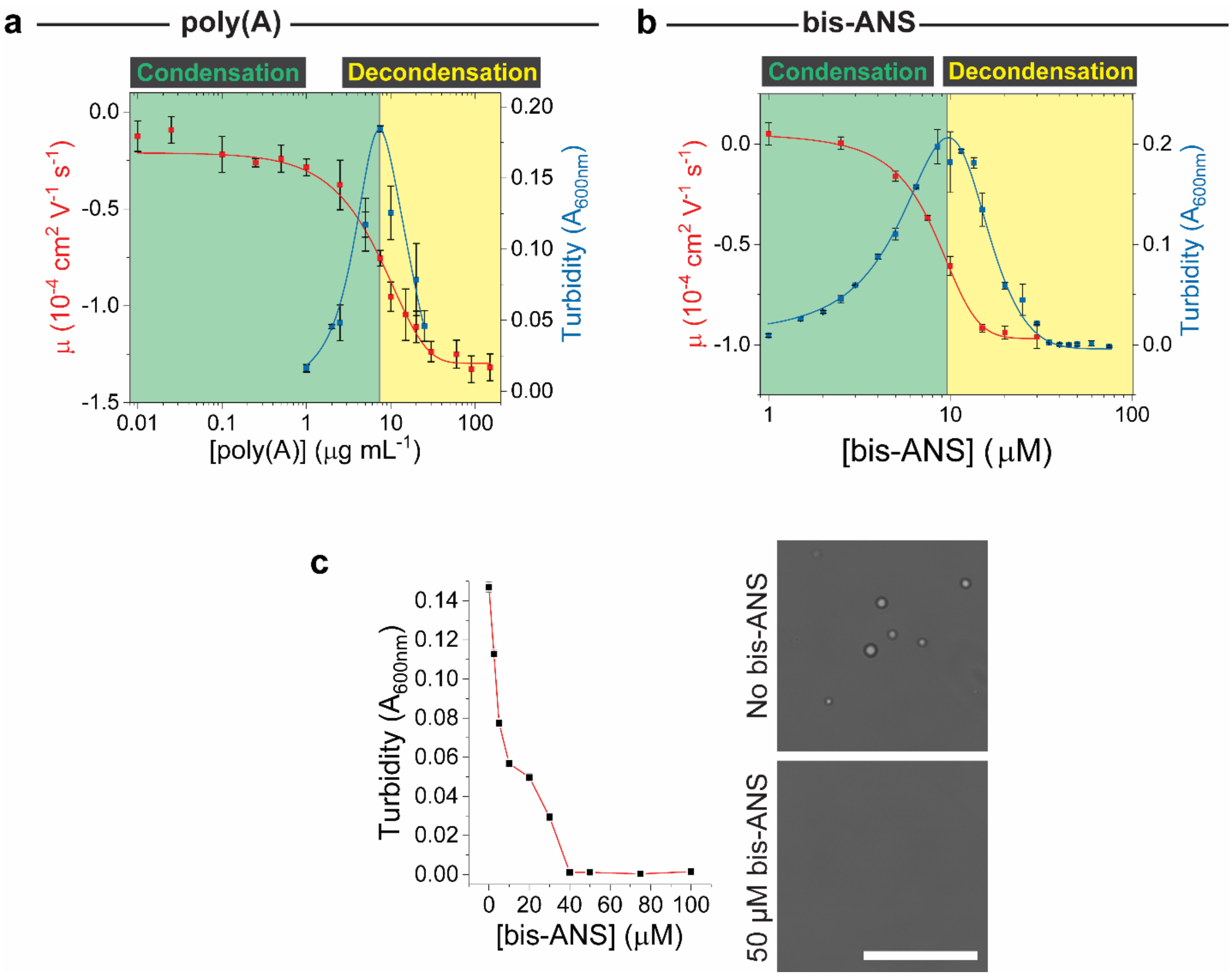

Supplementary Figure 4. **Charge-based interactions in poly(A) and bis-ANS systems.** Electrophoretic mobility (μ, red) of TDP-43 LCD (untagged) as a function of (**a**) poly(A) or (**b**) bis-ANS concentration (n = 3 separate preparations). The turbidity data (blue) representing the condensation-decondensation curve shown in Fig. 1c and Fig. 2a, respectively, are overlaid for reference. **c**, Turbidity data and representative microscopy images of TDP-43 LCD droplets in the presence of 7.5 μg mL^{-1} poly(A) and the abrogation of droplet formation by increasing concentrations of bis-ANS. Scale bar: 10 μm. All experiments depicted in this figure were performed in 20 mM potassium phosphate buffer, pH 6.0, using untagged protein at a concentration of 5 μM (*n* = 3 technical replicates). All data are presented as mean values +/- standard deviation.

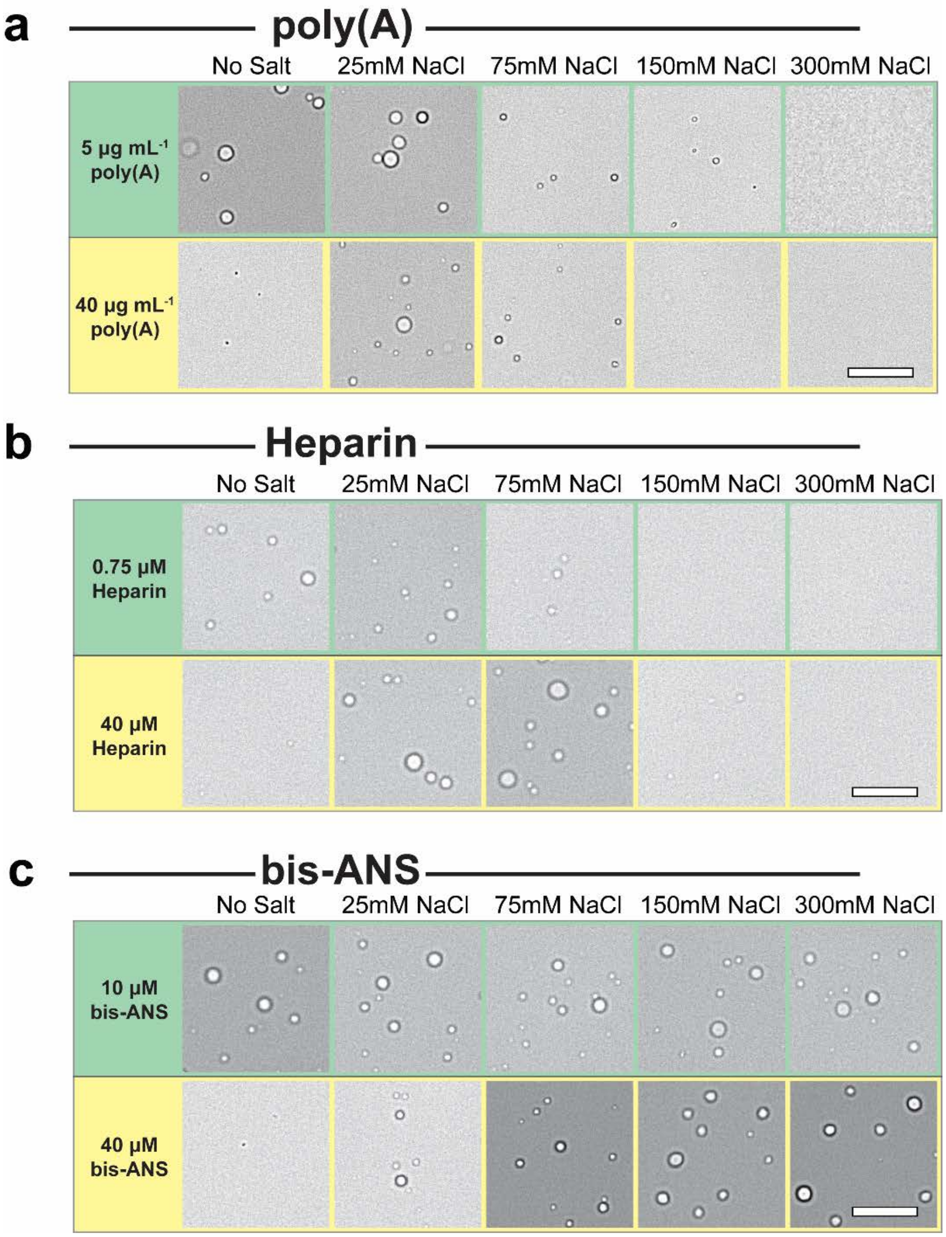

Supplementary Figure 5. **Effect of salt on LLPS induced by (a) poly(A), (b) heparin, and (c) bis-ANS.** Representative bright field images are shown for co-factor concentrations in the condensation regime (green panels) and in the decondensation regime (yellow panels), as originally defined in the absence of NaCl. Experiments were performed using 5 µM TDP-43 LCD in a pH 6 phosphate buffer containing varying concentrations of NaCl. Imaging was performed once per condition. Scale bar: 10 µm.

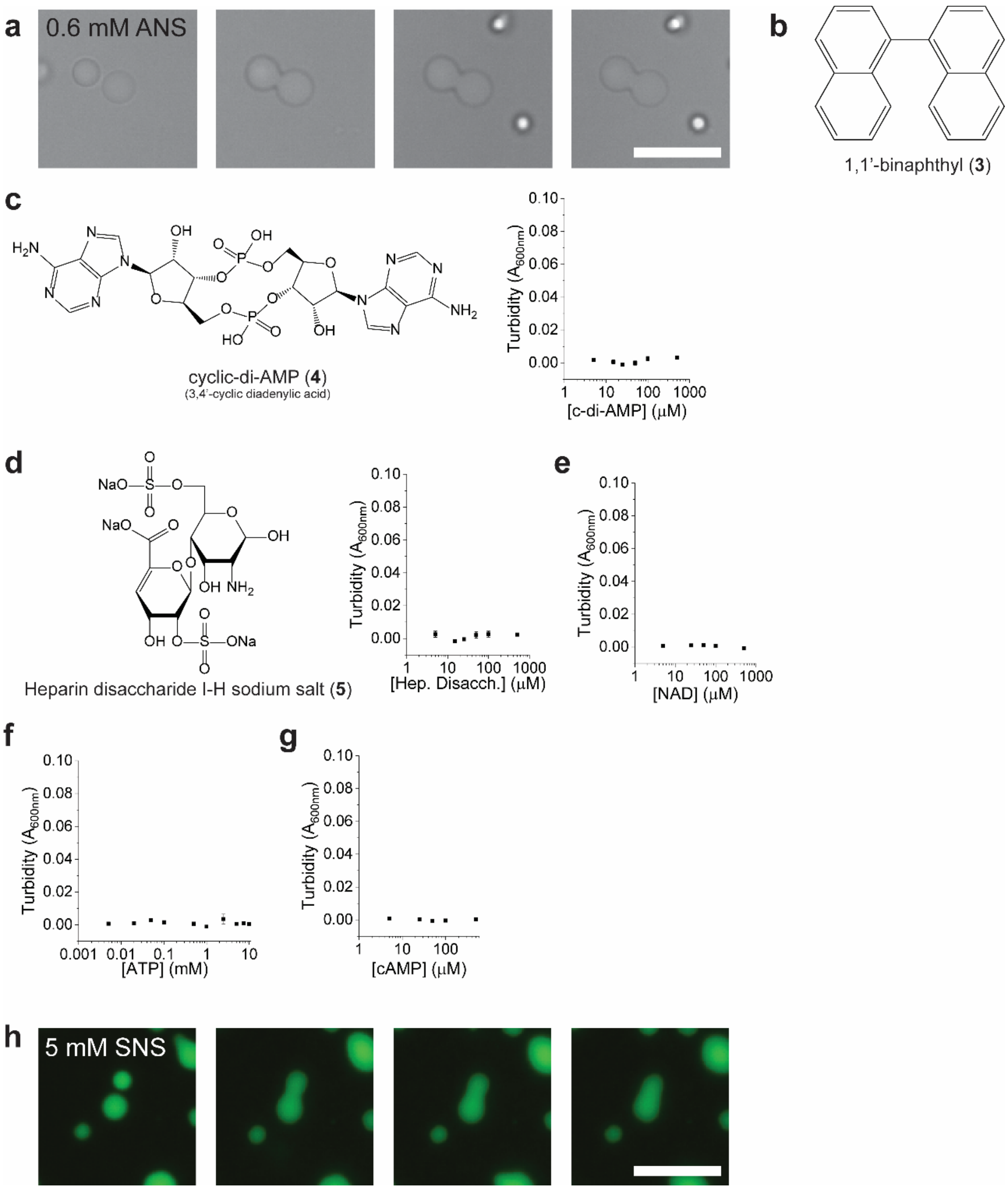

Supplementary Figure 6. **Testing other small molecules for LLPS induction capacity. a,** Time lapse microscopy showing a fusion event among droplets formed in the presence of ANS. Imaging to capture fusion events was performed once. **b**, Chemical structure of 1,1'-binaphthyl (**3**). **c,** Chemical structure and associated turbidity data in the presence of the bivalent cyclic-di-AMP (**3**). **d,** Chemical structure and associated turbidity data in the presence of the bivalent heparin disaccharide I-H. **e-g,** Turbidity data in the presence of varying concentrations of the bivalent nicotinamide adenine dinucleotide (NAD, **e**), monovalent ATP (**f**), and monovalent cAMP (**g**). **h**, Time-lapse microscopy showing a fusion event among droplets formed in the presence of SNS. Experiments were performed in a 20 mM potassium phosphate buffer, pH 6.0. Imaging to capture fusion events was performed once. All data are presented as mean values +/- standard deviation (*n* = 3 technical replicates). All scale bars: 10 μm.

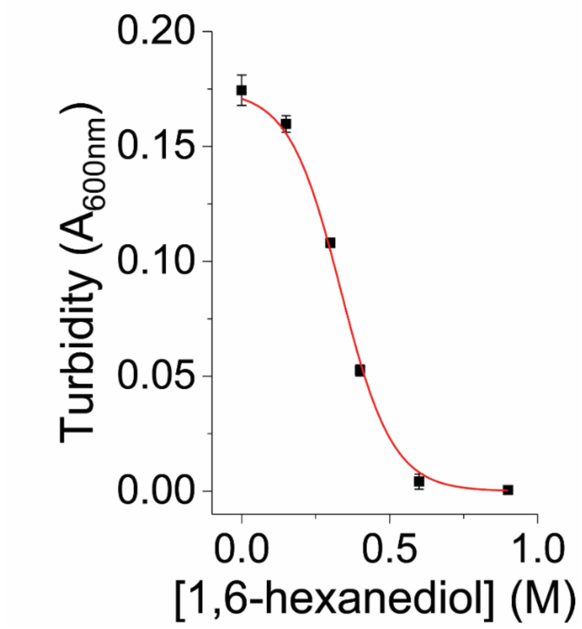

Supplementary Figure 7. **Effect of 1,6-hexanediol on TDP-43 LCD/bis-ANS LLPS.** Experiments were performed using 5 μM TDP-43 LCD and 10 μM bis-ANS in the presence of varying concentrations of 1,6-hexanediol and a pH 6 buffer containing 20 mM potassium phosphate. All data are presented as mean values +/- standard deviation (*n* = 3 technical replicates).

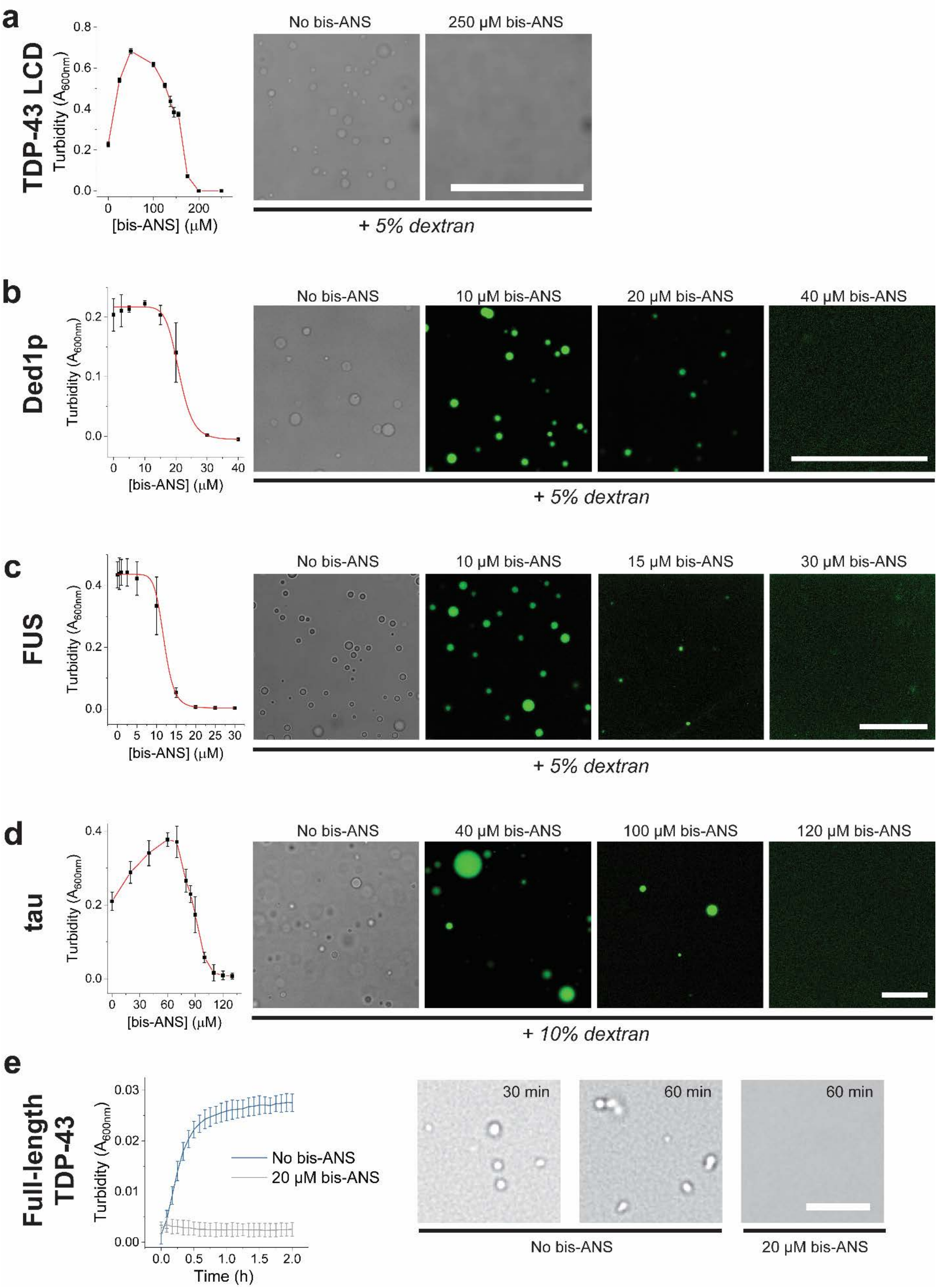
a
TDP-43 LCD
Turbidity (A600nm)
[bis-ANS] (μM)
No bis-ANS
250 μM bis-ANS
+ 5% dextran
b
Ded1p
No bis-ANS
10 μM bis-ANS
20 μM bis-ANS
40 μM bis-ANS
+ 5% dextran
c
FUS
No bis-ANS
10 μM bis-ANS
15 μM bis-ANS
30 μM bis-ANS
+ 5% dextran
d
tau
No bis-ANS
40 μM bis-ANS
100 μM bis-ANS
120 μM bis-ANS
+ 10% dextran
e
Full-length TDP-43
Time (h)
No bis-ANS
20 μM bis-ANS
30 min
60 min
60 min
No bis-ANS
20 μM bis-ANS

Supplementary Figure 8. **Bis-ANS prevents formation of homotypic droplets in a crowded environment. a-d**, Turbidity data and representative microscopy images depicting the formation of droplets from (**a**) TDP-43 LCD (20 μM), (**b**) Ded1p (2 μM), (**c**) FUS (5 μM), and (**d**) tau (10 μM) in the presence of dextran as a crowding agent and inhibition of this process by bis-ANS. Scale bars for (**a**-**d**): 25 μm. **e**, Turbidity data and representative microscopy images used to monitor the transient formation of droplets and subsequent aggregation of full-length TDP-43 (2.5 μM) in the presence or absence of 20 μM bis-ANS. This process is initiated by the addition of TEV protease that cleaves an MBP solubility tag from full-length TDP-43. Scale bar: 5 μm. All data are presented as mean values +/- standard deviation (*n* = 3 technical replicates).

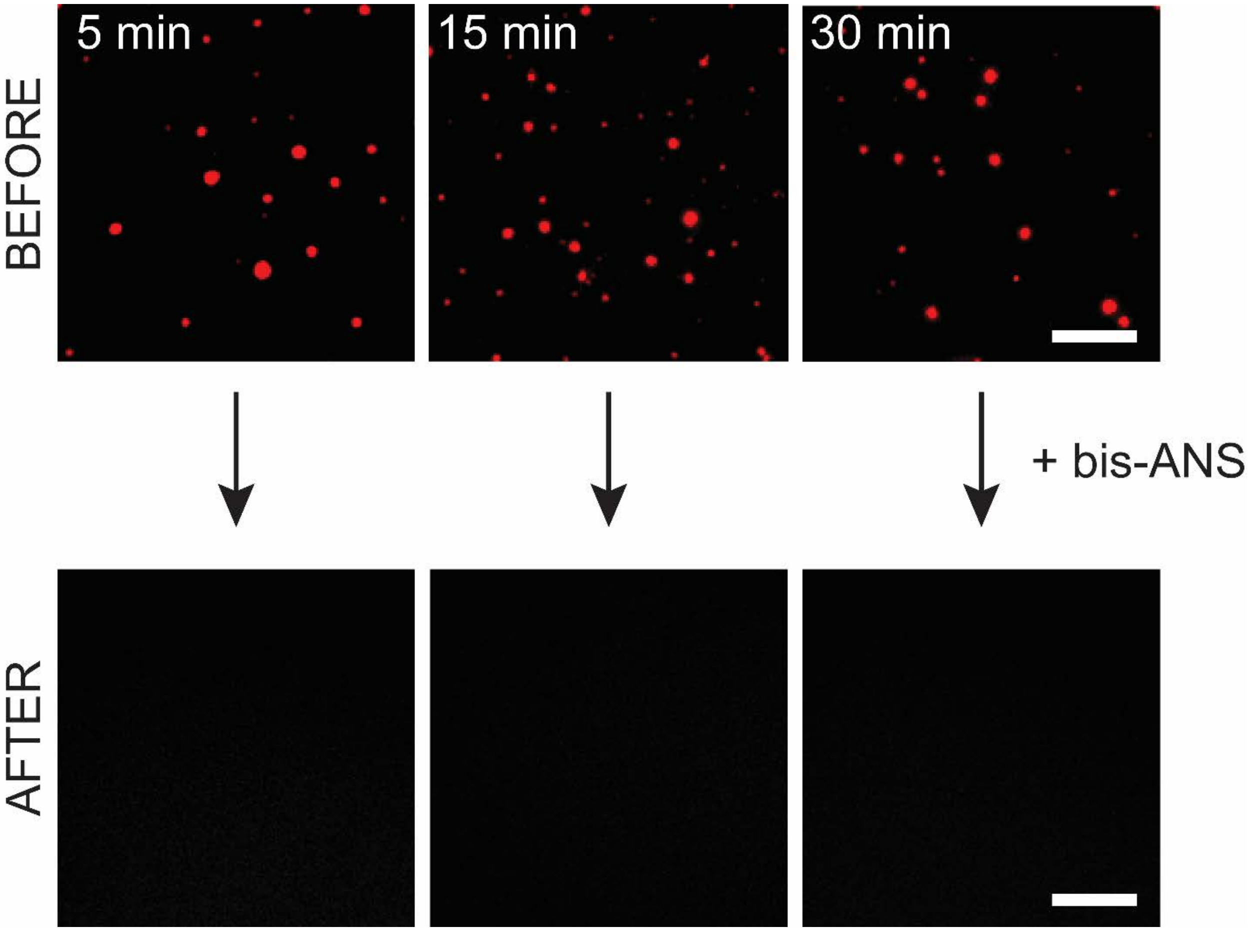

Supplementary Figure 9. **Preformed TDP-43 LCD droplets are disrupted by the addition of bis-ANS.** Representative fluorescence microscopy imaging before and after the addition of 250 μM bis-ANS to droplets formed by TDP-43 LCD (20 μM) in a pH 8 buffer containing 20 mM potassium phosphate, 5% dextran, and doped with 200 nM AlexaFluor 594-labeled TDP-43 LCD. Preformed droplets were incubated in buffer for 5, 15, or 30 minutes prior to the addition of bis-ANS. Images in bottom panels were obtained immediately after adding bis-ANS. Imaging was performed twice with similar results. Scale bars: 10 μm.

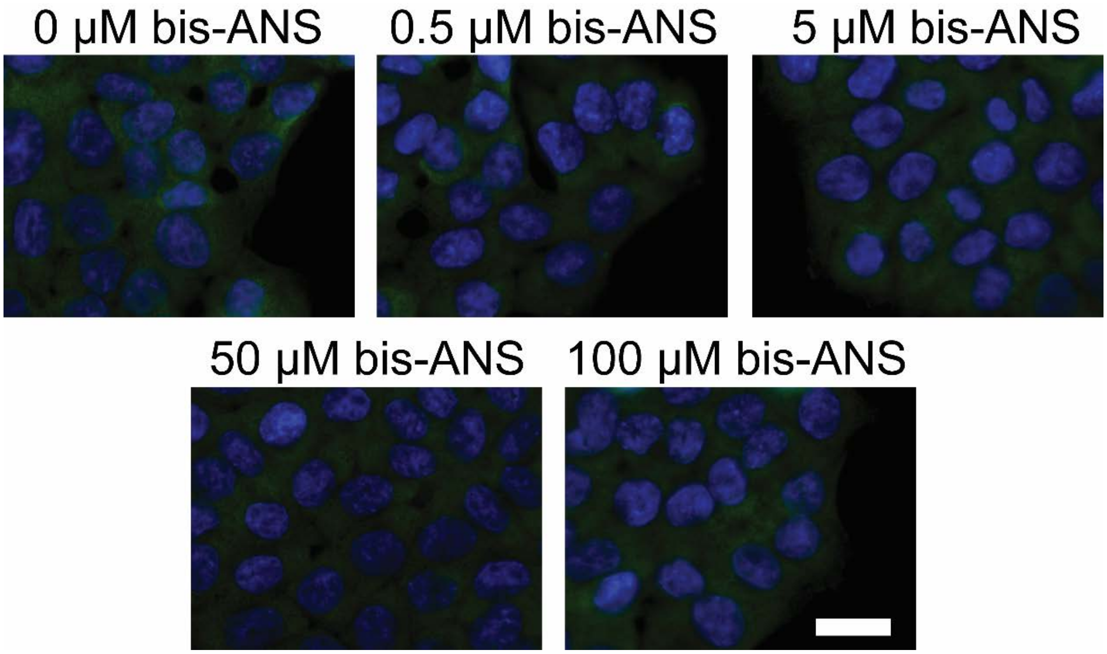

Supplementary Figure 10. **Treatment of HCT-116 cells with bis-ANS.** HCT-116 cells were treated with various concentrations of bis-ANS for 2 h, then fixed and stained with DAPI (blue) and an antibody against G3BP (green). Diffuse G3BP staining indicates that bis-ANS alone does not induce G3BP+ stress granules. Scale bar represents 20 μm.
