# Supplementary material for: Small molecules as potent biphasic modulators of protein liquid-liquid phase separation
Source: Nat Commun. 2020 Nov 4;11:5574. doi: 10.1038/s41467-020-19211-z (PMC7643064; doi:10.1038/s41467-020-19211-z)
Supplement: Supplementary file 3 — Reporting Summary [file 41467_2020_19211_MOESM3_ESM.pdf]

nature research

Corresponding author(s): Witold K. Surewicz

Last updated by author(s): Sep 23, 2020

Reporting Summary

Nature Research wishes to improve the reproducibility of the work that we publish. This form provides structure for consistency and transparency in reporting. For further information on Nature Research policies, see Authors & Referees and the Editorial Policy Checklist.

Statistics

For all statistical analyses, confirm that the following items are present in the figure legend, table legend, main text, or Methods section.

n/a	Confirmed	
☐	☒	The exact sample size (*n*) for each experimental group/condition, given as a discrete number and unit of measurement
☐	☒	A statement on whether measurements were taken from distinct samples or whether the same sample was measured repeatedly
☒	☐	The statistical test(s) used AND whether they are one- or two-sided<br>*Only common tests should be described solely by name; describe more complex techniques in the Methods section.*
☒	☐	A description of all covariates tested
☒	☐	A description of any assumptions or corrections, such as tests of normality and adjustment for multiple comparisons
☐	☒	A full description of the statistical parameters including central tendency (e.g. means) or other basic estimates (e.g. regression coefficient) AND variation (e.g. standard deviation) or associated estimates of uncertainty (e.g. confidence intervals)
☒	☐	For null hypothesis testing, the test statistic (e.g. *F*, *t*, *r*) with confidence intervals, effect sizes, degrees of freedom and *P* value noted<br>*Give P values as exact values whenever suitable.*
☒	☐	For Bayesian analysis, information on the choice of priors and Markov chain Monte Carlo settings
☒	☐	For hierarchical and complex designs, identification of the appropriate level for tests and full reporting of outcomes
☒	☐	Estimates of effect sizes (e.g. Cohen's *d*, Pearson's *r*), indicating how they were calculated

Our web collection on statistics for biologists contains articles on many of the points above.

Software and code

Policy information about availability of computer code

Data collection	Tecan SPARKControl (ver. 2.3), BZ-X Viewer (Keyence, ver. 1.3.1.1), Malvern Zetasizer Software (ver. 7.13), Leica Application Suite X (3.3.0.16799), www.chemspider.com; https://pubchem.ncbi.nlm.nih.gov; ChemDraw Professional (ver. 17.0)
Data analysis	BZ-X Analyzer (Keyence, ver. 1.3.1.1), Excel (ver. 2013, 2017), Origin (ver. 2017, 2019), ImageJ (ver. 1.53c)

For manuscripts utilizing custom algorithms or software that are central to the research but not yet described in published literature, software must be made available to editors/reviewers. We strongly encourage code deposition in a community repository (e.g. GitHub). See the Nature Research guidelines for submitting code & software for further information.

Data

Policy information about availability of data

All manuscripts must include a data availability statement. This statement should provide the following information, where applicable:

- Accession codes, unique identifiers, or web links for publicly available datasets
- A list of figures that have associated raw data
- A description of any restrictions on data availability

The data generated in this study are available from the corresponding authors upon reasonable request. The source data underlying Fig.1-7 and Supplementary Fig. 1,2,4-8 are provided as a Source Data file.

Field-specific reporting

Please select the one below that is the best fit for your research. If you are not sure, read the appropriate sections before making your selection.

☒ Life sciences ☐ Behavioural & social sciences ☐ Ecological, evolutionary & environmental sciences

For a reference copy of the document with all sections, see nature.com/documents/nr-reporting-summary-flat.pdf

Life sciences study design

All studies must disclose on these points even when the disclosure is negative.

Sample size	No statistical analysis was used to predetermine sample size because no group comparisons were made. Consistent with prior studies performing similar assays (PMID 28819146, PMID 32499559, PMID 27545621, PMID 30814253), each experiment was performed with a minimum of three technical replicates.
Data exclusions	Data were not excluded from the analyses.
Replication	Reproducibility was validated by repeating experiments using independent sample preparations (as noted in figure legends, where relevant) in addition to technical replicates for each individual preparation. For recombinant protein studies, independent preparations are defined by separate purification batches and, for cellular studies, independent preparations (biological replicates) are defined by separate passages of cells.
Randomization	Randomization was not relevant to experiments involving recombinant protein as samples were not assigned to specific groups. For cellular studies, images were quantified in the absence of treatment group identifiers.
Blinding	Blinding was not applicable to this study. The authors who performed each experiment also analyzed the data.

Reporting for specific materials, systems and methods

We require information from authors about some types of materials, experimental systems and methods used in many studies. Here, indicate whether each material, system or method listed is relevant to your study. If you are not sure if a list item applies to your research, read the appropriate section before selecting a response.

Materials & experimental systems

n/a	Involved in the study	
☐	☒	Antibodies
☐	☒	Eukaryotic cell lines
☒	☐	Palaeontology
☒	☐	Animals and other organisms
☒	☐	Human research participants
☒	☐	Clinical data

Methods

n/a	Involved in the study	
☒	☐	ChIP-seq
☒	☐	Flow cytometry
☒	☐	MRI-based neuroimaging

Antibodies

Antibodies used	Mouse anti-G3BP (Sigma; WH0010146M1), anti-mouse CF488 (Sigma; SAB4600237)
Validation	For mouse anti-G3BP (Sigma; WH0010146M1), validation was performed by manufacturer and deemed "suitable" for immunofluorescence (https://www.sigmaaldrich.com/catalog/product/sigma/wh0010146m1). For anti-mouse CF488 secondary antibody (Sigma; SAB4600237), validation for immunofluorescence was performed by manufacturer (https://www.sigmaaldrich.com/catalog/product/sigma/sab4600237).

Eukaryotic cell lines

Policy information about cell lines

Cell line source(s)	ATCC (CCL-247)
Authentication	Authentication performed by STR profiling by distributor
Mycoplasma contamination	PCR testing indicated cell lines were free of mycoplasma contamination
Commonly misidentified lines (See ICLAC register)	No commonly misidentified cell lines were used in this study.
